# Supplementary material for: The shared genetic architecture and evolution of human language and musical rhythm
Source: bioRxiv. 2023 Nov 2:2023.11.01.564908. Preprint. [Version 1] doi: 10.1101/2023.11.01.564908 (PMC10634981; doi:10.1101/2023.11.01.564908)
Supplement: Supplement 1 [file media-1.pdf]

Supplementary Materials for

The shared genetic architecture and evolution of human language and musical rhythm

Gökberk Alagöz[#1], Else Eising[1], Yasmina Mekki[2,3], Giacomo Bignardi[1,4], Pierre Fontanillas[5], 23andMe Research Team, Michel G. Nivard[6], Michelle Luciano[7], Nancy J. Cox[3], Simon E. Fisher[#*1,8], Reyna L. Gordon[#*2,3,9,10]

[1] Language and Genetics Department, Max Planck Institute for Psycholinguistics, 6500 AH Nijmegen, The Netherlands
[2] Department of Otolaryngology - Head & Neck Surgery, Vanderbilt University Medical Center, Nashville, TN, USA.
[3] Vanderbilt Genetics Institute, Vanderbilt University Medical Center, Nashville, TN, USA.
[4] Max Planck School of Cognition, Leipzig, Germany
[5] 23andMe, Inc., Sunnyvale, CA, USA
[6] Department of Biological Psychology, Vrije Universiteit, Amsterdam, the Netherlands.
[7] Department of Psychology, University of Edinburgh, Edinburgh, UK.
[8] Donders Institute for Brain, Cognition and Behaviour, Radboud University, 6500 HB Nijmegen, The Netherlands
[9] Vanderbilt Brain Institute, Vanderbilt University, Nashville, TN, USA.
[10] The Curb Center, Vanderbilt University, Nashville, TN, USA.

[#] Correspondence: goekberk.alagoez@mpi.nl, simon.fisher@mpi.nl, reyna.gordon@alumni.usc.edu
*** These authors contributed equally to this work.**

This PDF file includes:
Supplementary Figures 1 to 8

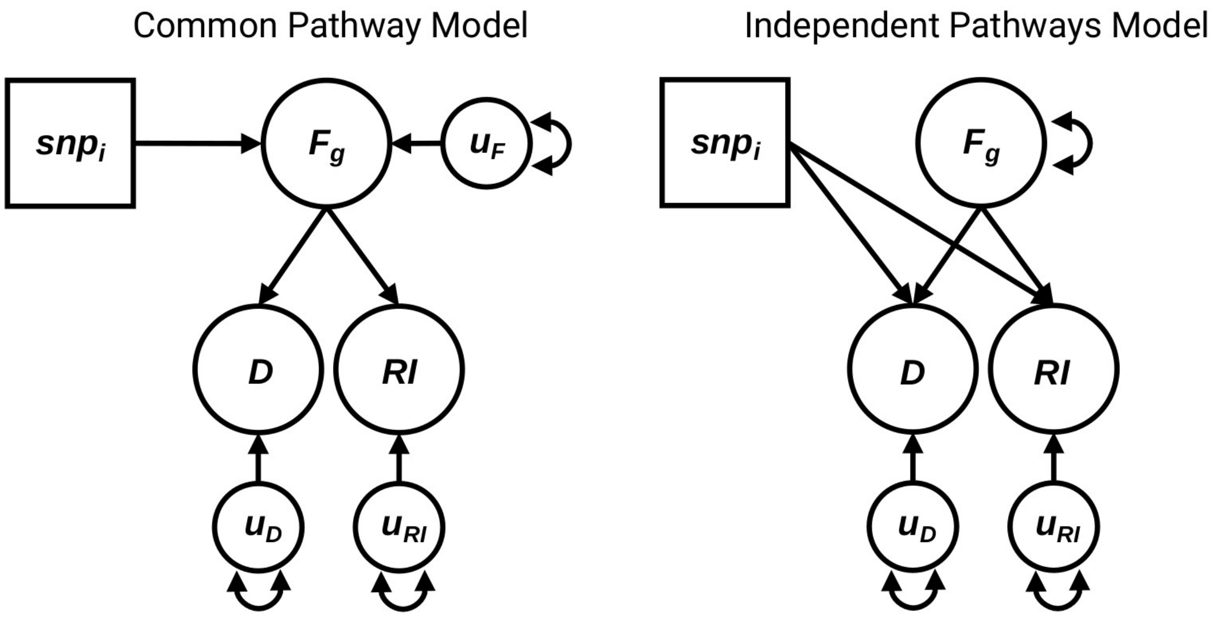

Supplementary Figure 1: Genomic SEM model diagrams for common pathway and independent pathways models. The models were used in the mvGWAS for the common factor and independent factor association analyses. D: dyslexia, RI: rhythm impairment, F_g: Common factor, u_D: residual variance of dyslexia, u_{RI}: residual variance of rhythm impairment, u_F: residual variance of the common factor, snp_i: i^{th} SNP regression.

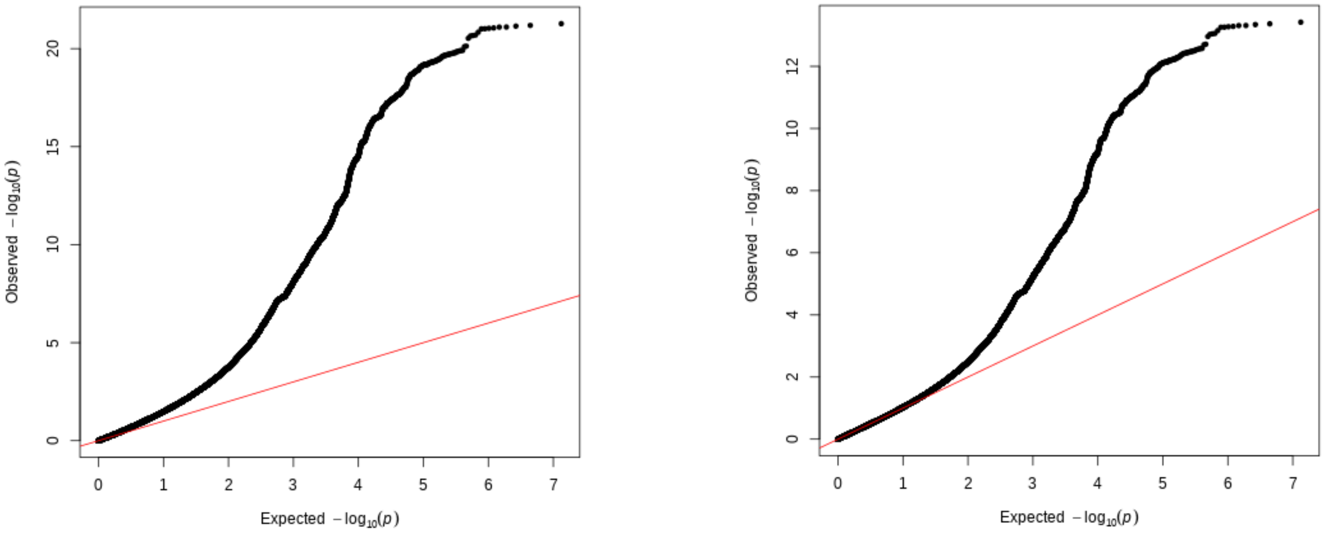

Supplementary Figure 2: QQ plots of F_{gRI-D} mvGWAS summary statistics *prior to* (left) and *after* (right) GC correction.

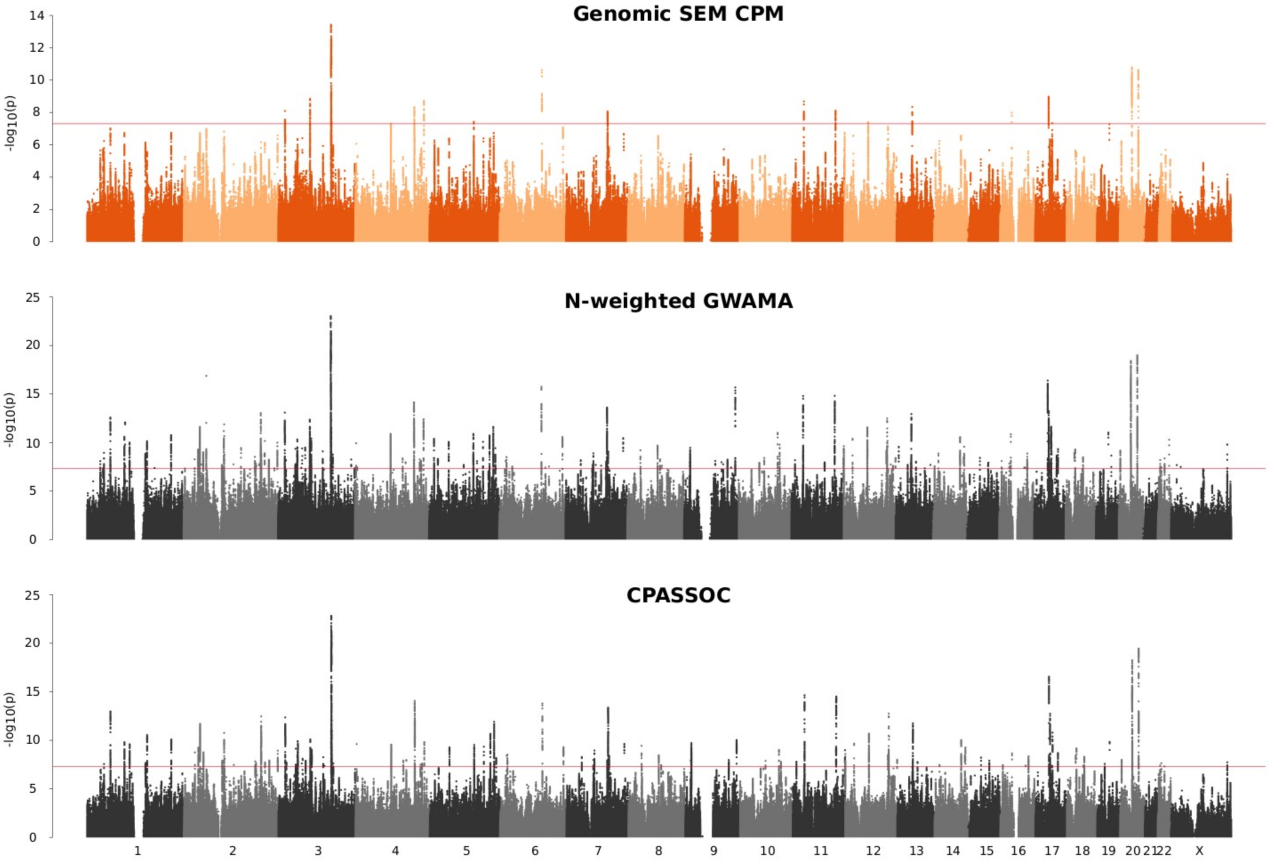

Supplementary Figure 3: Manhattan plots for Genomic SEM CPM (F_{gRI-D}), N-weighted GWAMA, and CPASSOC. The red lines correspond to the genome-wide significance threshold ($P<5\times10^{-8}$).

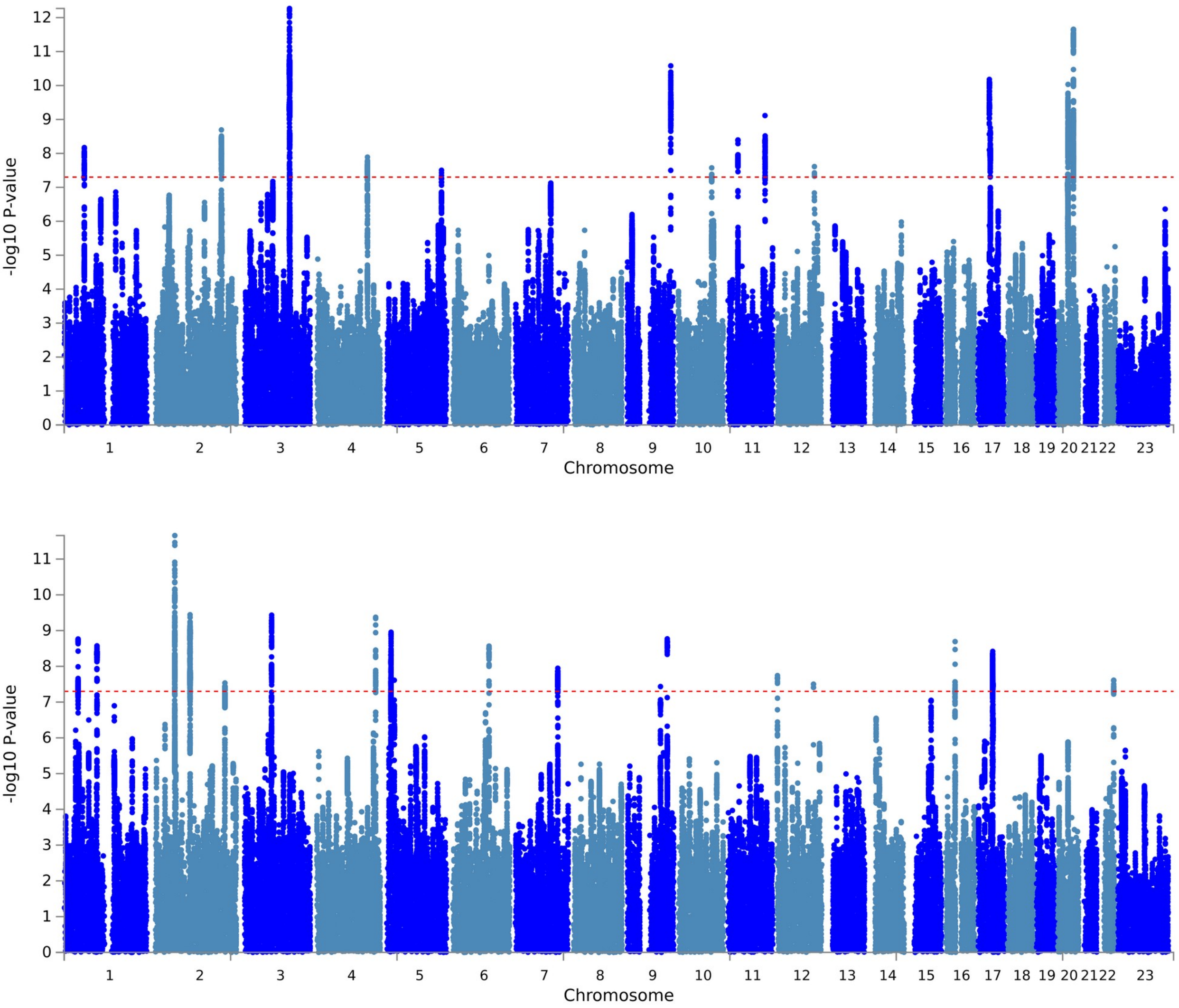

Supplementary Figure 4: Manhattan plots for the independent factors of dyslexia (top) and rhythm impairment (bottom). The red lines correspond to genome-wide significance threshold ($P<5\times10^{-8}$).

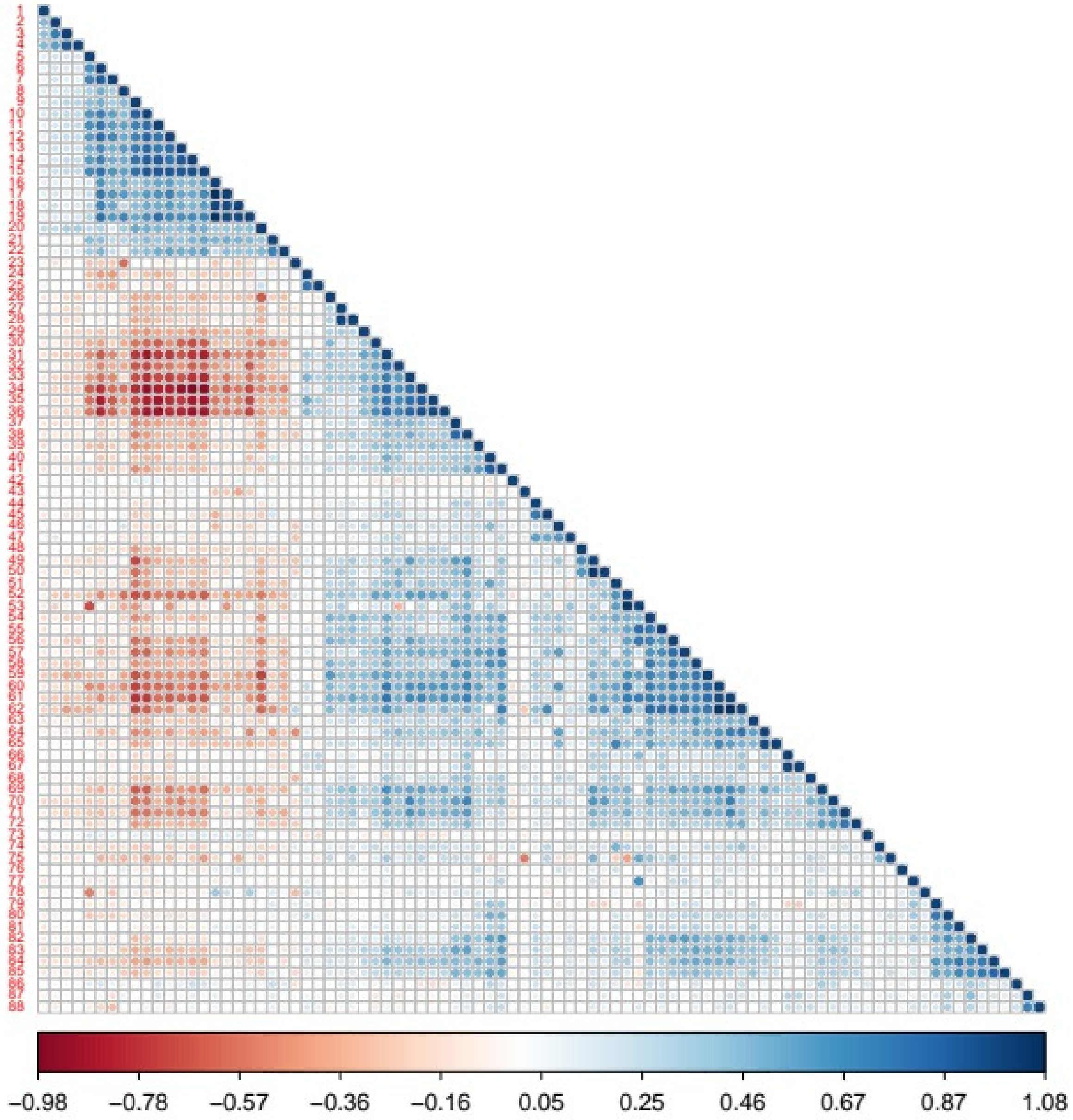

Supplementary Figure 5: Genetic correlations (r_g) among 88 traits that are significantly correlated either with dyslexia or rhythm. Genetic correlations were estimated using LDSC. Supplementary Table 13 provides the list of traits included.

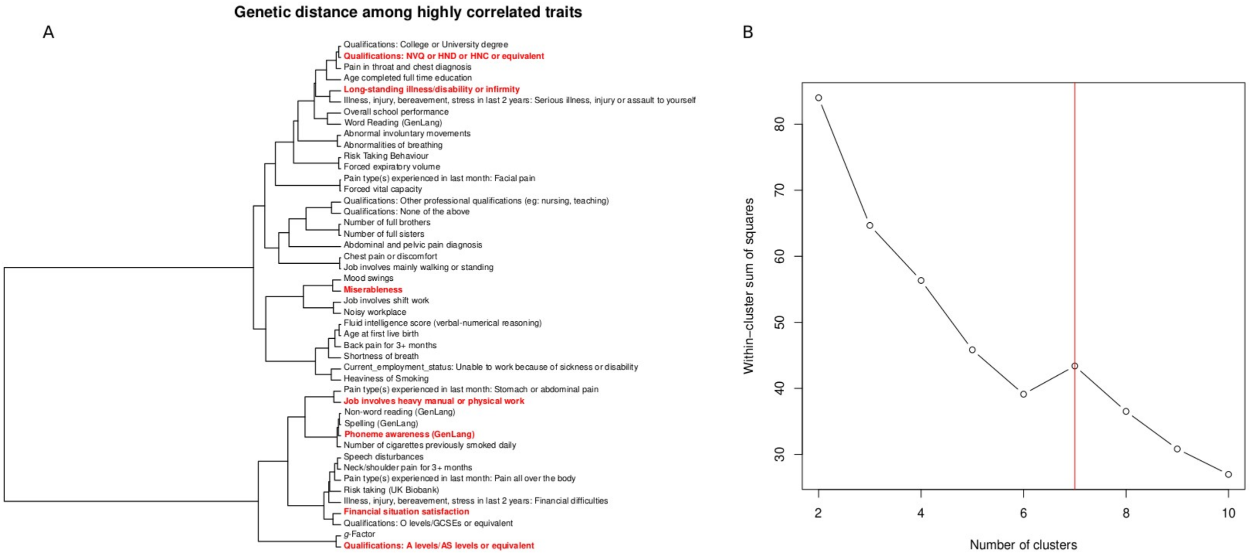

Supplementary Figure 6: (A) Dendrogram showing the hierarchical clustering of highly genetically correlated ($|r_g|$>0.80) traits. (B) Knee-point algorithm identified 7 representative clusters with. For each cluster, one representative trait (shown in bold red) was used in the genetic correlation analysis with the $F_{gRI\text{-}D}$.

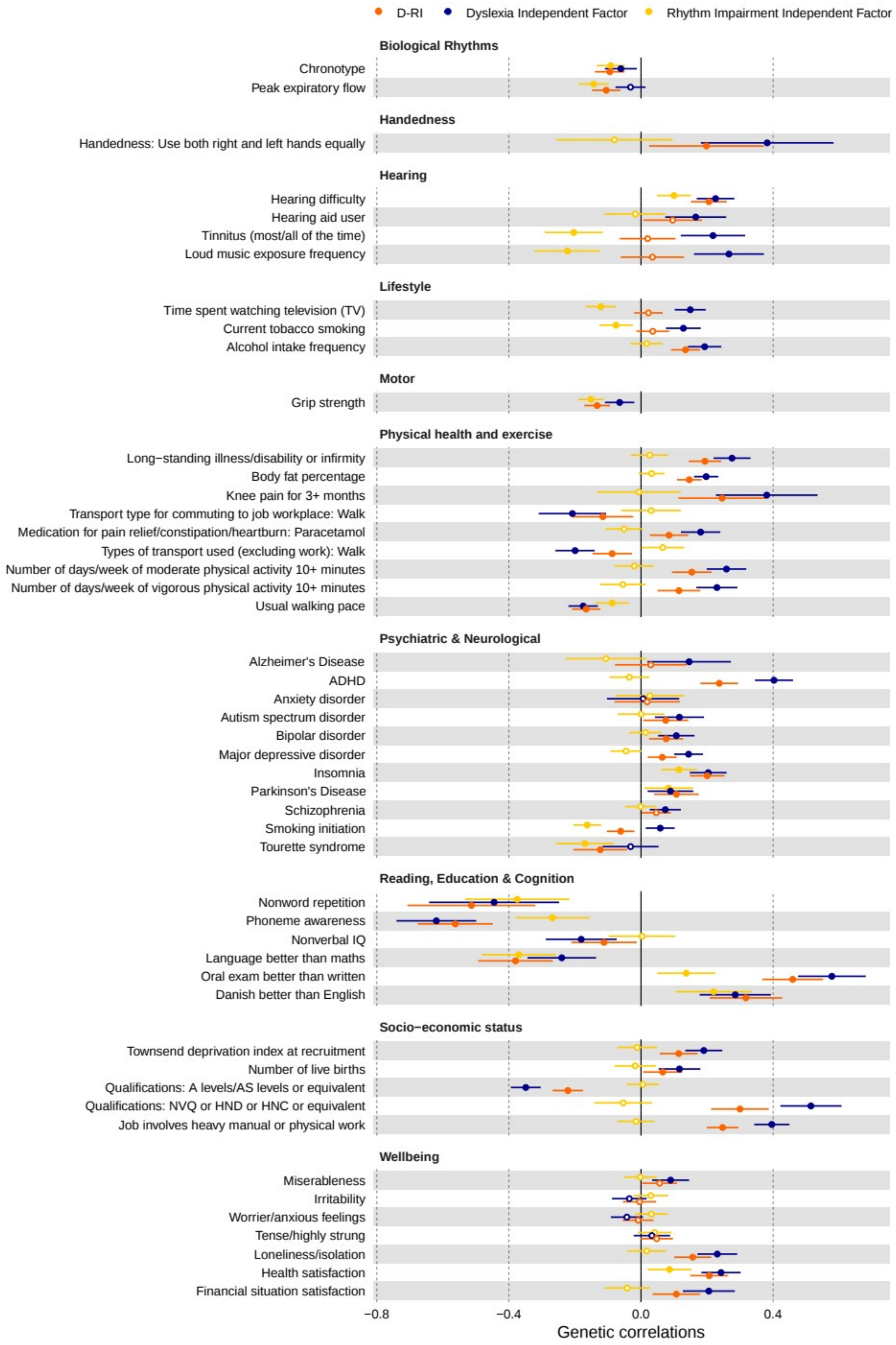

Supplementary Figure 7: Genetic correlations (r_g) between the selected 49 traits and F_{gRI-D} (D-RI) (orange) and dyslexia (blue) and rhythm (yellow) independent factors. Genetic correlations were estimated using LDSC. Full circles indicate significant correlations (P<0.05). Error bars represent standard errors.

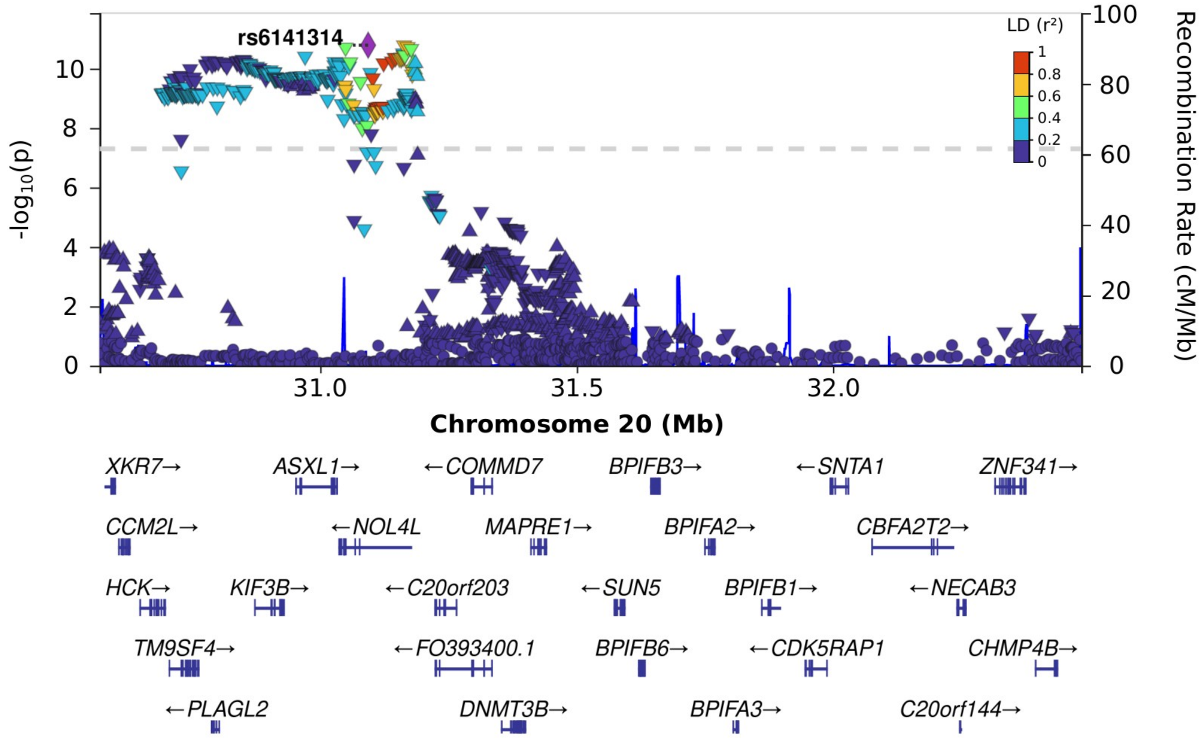

Supplementary Figure 8: LocusZoom plot of chr20: 30,569,660-32,484,506 locus, the region which is identified by local genetic correlation analysis of F_{gRI-D} and Superior Longitudinal Fasciculus I. P-values represent F_{gRI-D} mvGWAS significance. Direction of the triangles represent effect directions. LD (r^2) levels with rs6141314 are represented in colours. Grey dash-line indicate genome-wide significance level ($P<5x10^{-8}$).
